# The Effect of Exogenously Applied Dicarboxylic Acids (Photon) on the Maize (*Zea mays*) Metabolite Profile

**DOI:** 10.1002/cbdv.202501243

**Published:** 2025-07-22

**Authors:** Mhlonipheni Msomi, Noluyolo Nogemane, Garland More, Gerhard Prinsloo

**Affiliations:** ^1^ Department of Agriculture and Animal Health University of South Africa Florida Science Campus Johannesburg Gauteng South Africa; ^2^ CAES Laboratories, College of Agriculture and Environmental Sciences University of South Africa Johannesburg Gauteng South Africa

**Keywords:** ^1^H‐NMR‐based metabolomics, antioxidant enzymes, dicarboxylic acids, maize, metabolite profiling

## Abstract

Maize is an important crop worldwide with approximately 1200 million tons consumed and a market size worth $143 billion. Environmental stress influence production of crops and mitigating these ensures optimum production. Dicarboxylic acids are known as emulsifying agents that mitigate various stresses in plants. Previous reports have shown that mixtures of dicarboxylic acids like azelaic and sebacic acid are equally important for plants and have improved plant resistance to stress by directly or indirectly affecting metabolic components. In this study, maize plants in their six‐leaf (V6) growth stage were sprayed with a mixture of dicarboxylic acids (photon) to investigate the effect of priming on the metabolic profile of maize. A nontargeted ^1^H‐NMR‐based metabolic approach was used to determine the metabolic responses of maize sprayed with photon and grown under the same conditions. Leaf samples were collected at various time points (1 h, 2 h, 12 h, 24 h, 1 week, 2 weeks, and 3 weeks) after application of photon. Orthogonal partial least squares–discriminant analysis (OPLS‐DA) revealed various metabolites that were involved in tolerance to plant stress. A significant increase in salicylic acid (SA) and azelaic acid (AzA) was observed in photon‐treated samples compared to the controls, and it was also responsible for an increase in amino acids (GABA, alanine, and asparagine) and sugars (sucrose, maltose, and trehalose). In addition, malate and aconitate accumulated after 2 h of treatment and were found to be present in higher concentrations in the treated maize leaf extracts. Since SA was identified as one of the major compounds in treated plants, the enzymatic antioxidants (SOD, CAT, and SOD) were further evaluated. Limited SOD and CAT activity at concentrations ranging from 16.63 to 3.91 µg/mL was detected. The POD showed the lowest activity with no significant differences between photon‐treated and untreated extracts. The results indicate that maize plants treated with photon showed appreciable changes in various metabolites, although no significant priming effect on antioxidant enzyme activities was detected. Therefore, the priming effect is proposed to involve the SAR in maize.

## Graphical abstract

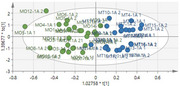

## 1 Introduction

Maize (*Zea mays*) is a globally consumed crop that is currently facing an increasing demand due to an upward consumption trend. Its adaptability enables the production of several products, including bioethanol, animal feed, syrup, oil, and cornmeal, all of which enhance the grain's increasing appeal [1]. As a cereal crop, it is mainly grown in semiarid regions of the world where seasonal environmental stressors are prevalent [2]. Successful production is essential to meet the growing population for sustainable food production.

Maize contains a wide variety of metabolites that are crucial for plant development, defense signaling, and adaptation to a variety of environmental stresses [3, 4]. These metabolites accumulate in plants when exposed to stress such as various elicitors or signal molecules [5, 6]. The plants are subsequently conditioned to enhance their resistance to biotic and abiotic stresses. Variations in the plant's physicochemical environment, such as temperature or soil composition, have a direct effect on the plant's metabolism [7]. Plant metabolism must be rapidly, flexibly, and coordinately regulated in response to fluctuating environments, which can sometimes be drastically changed in an order of seconds [8]. Consequently, understanding the metabolite profile of maize, the influence of priming agents, and their impact on biochemical features prior to and following exposure to stressors, remains pivotal in guiding plant resistance to both biotic and abiotic stress [9, 10]. In addition to metabolites, plants also employ other mechanisms to maintain homeostasis by production of antioxidants. Antioxidants such as superoxide dismutase (SOD), catalase (CAT), glutathione ascorbate (GPX), ascorbate peroxidase (APX), and peroxidase (POD) scavenge excess ROS to maintain physiological functions [11].

Photon is a blend of saturated dicarboxylic acids that is recognized for enhancing crop productivity relative to untreated crops, preparing them for harsh environmental conditions [12]. This dicarboxylic formulation containing suberic acid, azelaic acid (AzA), and sebacic acid, is known to influence stress resistance of crops. Among these crops, maize has demonstrated positive results following photon treatment [12]. However, to date limited information on the mechanism of action is available and mapping the metabolite changes will elucidate the strategies that plants use to enhance resistance to biotic and abiotic stressors.

In plant research, metabolomics has proven to be a powerful tool for identifying phenotypic effects of abiotic and biotic stresses [13], and a metabolomics‐based approach was used to gain insight into the effect of photon on the plant response. Metabolic profiling has emerged as one of the most significant breakthroughs in recent years for obtaining reliable directions about plants [14]. Methods such as hyphenated mass spectrometry (MS) and nuclear magnetic resonance (NMR) spectroscopy are used to analyze the maize metabolome [15]. As one of the metabolomic technologies, NMR spectroscopy contributes significantly to the profiling of metabolites for various plant responses and tolerance mechanisms. In addition, it is one of the most effective complementary techniques employed for identifying metabolites in plant extracts.

This study aimed to investigate the effect of dicarboxylic acids (photon) on the metabolome of *Z. mays* leaf extracts using ^1^H‐NMR‐based metabolomics. In addition, the enzymes SOD, CAT, and POD were evaluated to determine whether photon influenced the antioxidant system of maize by evaluating the antioxidant activity (SOD, CAT, and POD) of the leaf extracts of *Z. mays*.

## 2 Materials and Methods

### 2.1 Experimental Site

The study was conducted at the University of South Africa, Science Campus, Gauteng Province of South Africa (26°10′30″ S; 27°55′22.8″ E) during the 2021–2022 period.

### 2.2 Plant Material Treatment

The PAN3A‐173 maize seed variety from Pannar was grown at the University of South Africa, Science Campus, Gauteng province of South Africa during the 2021–2022 period. A total of 32 pots (30 cm) with 16 replicates of treated plants were randomized and another 16 replications served as a positive control. All plants were grown in a polypropylene shade net with a 70% shade factor. A 50% mixture of naturally occurring dicarboxylic acids—suberic acid, AzA, and sebacic acid (Crop Microclimate Management Inc., Apex, North Carolina, USA) was sprayed at six‐leaf (V6) stage 2 h after sunrise using an 8 L hand‐held pressure sprayer (Ningbo, China), prepared by adding 16 mg/L of water. After spraying, leaves were harvested at various time intervals (1 h, 2 h, 12 h, 24 h, 1 week, 2 weeks, 3 weeks) and immediately submerged in liquid nitrogen (N_2_) (Afrox Wadeville Gas and Gear, Germiston, South Africa) and crushed using a cooled mortar and pestle to stop further metabolic activity. The leaves were then freeze‐dried for seven days, and the dried samples were stored in centrifuge tubes at −80°C until use.

### 2.3 ^1^H‐NMR Analysis

#### 2.3.1 Sample Preparation and Extraction Procedure

A direct extraction method using deuterated solvents (Merck (Pty) Ltd., Modderfontein, South Africa) was used. Metabolites were extracted by adding deuterated methanol/water (1:1) to 50 mg of fine powder from each sample in 2 mL Eppendorf tubes (Sigma‐Aldrich, Johannesburg, South Africa). The weighed samples were dissolved in 750 µL CH_3_OH‐d_4_ and a potassium phosphate buffer (750 µL, pH 6) in D_2_O containing 0.01% internal reference standard 3‐(trimethylsilyl) propionic‐2,2,3,3‐d_4_ acid sodium salt (TSP). At room temperature, the mixture was vortexed for 1 min to homogenize it, then ultrasonicated for 20 min to improve extraction, after which the supernatant was transferred to a Norell 5 mm NMR tube 9 (Sigma‐Aldrich) for spectroscopic analysis using a ^1^H‐NMR 600 MHz spectrometer (Varian Inc., CA, USA) recording 32 scans.

#### 2.3.2 Data Preprocessing

First, ^1^H‐NMR spectra were preprocessed using MestReNova (Mestrelab Research 14.2, Santiago de Compostela, Spain). Normalization, phase correction, baseline correction, and referencing to TSP standard (0.0) were applied to the acquired NMR spectra.

#### 2.3.3 Data Reduction of the ^1^H‐NMR Spectra

The ^1^H‐NMR spectra (0.0–10.0 ppm) were automatically data compressed to 250 integral segments with a width of 0.04 ppm to lessen the impact of chemical shift fluctuation caused by minute pH variations. To avoid the impacts of the residual water peak, the region *δ* 4.6–5.0 was excluded from the analysis. Similarly, 3.28–3.32 ppm range was removed as it represented the solvent methanol. Each segment consisted of the integral of the NMR region to which it was associated. Excel was then used, where for each sample the spectral integrals were scaled to the total intensity of the integrated spectrum. The samples were then imported into the SIMCA software package (version 15.0.2, Umetrics, Umea, Sweden) for multivariate analysis.

#### 2.3.4 Multivariate Data Analysis

The data analysis step is critical in metabolomics, metabolite fingerprinting, and metabolic profiling, which emphasizes the importance of bioinformatics [16]. To improve interpretation, the bucketed data was analyzed using multivariate principal component analysis (PCA) and orthogonal partial least squares–discriminant analysis (OPLS‐DA) multivariate statistical methods. The data was pareto scaled and validation of the model was tested using a 100 permutation test.

#### 2.3.5 Metabolite Annotation

The availability and quality of databases are crucial for metabolite annotation and identification [17]. In this study, Chenomx NMR suite 8.6 (evaluation edition) and the Human Metabolome Database (HMDB), as well as published literature were used for metabolite annotation.

#### 2.3.6 UPLC Analysis

LC–MS analysis was conducted to confirm the compounds annotated by the metabolomic analysis. A Waters Premier UPLC coupled in series to a Waters SYNAPT XS HDMS mass spectrometer was used to generate accurate mass data. Optimization of the chromatographic separation was done utilizing a Waters HSS T3 C18 column (150 mm × 2.1 mm, 1.8 µm) and the column temperature controlled at 60°C. A binary solvent mixture was used consisting of water (Eluent A) containing 10 mM formic acid (natural pH of 2.4) and acetonitrile (Eluent B) containing 10 mM formic acid. The initial conditions were 100%A at a flow rate of 0.4 mL/min and were maintained for 1 min, followed by a linear gradient to 10%A at 16 min. The conditions were kept constant for 2 min and then changed to the initial conditions. The runtime was 20 min and the injection volume varied between 1 and 10 µL. Samples were kept cool at 8°C in the Waters Sample Manager during the analysis.

#### 2.3.7 TOF Mass Spec Analysis

The SYNAPT XS mass spectrometer was used in sensitivity mode and operated with an electrospray probe to enable detection of all ESI‐compatible compounds. Leucine enkephalin (50 pg/mL) was used as reference calibrant (Lock Mass) to obtain typical mass accuracies between 0.1 and 2 mDalton (mDa). The mass spectrometer was operated in both ESI positive and negative modes with a capillary voltage of 0.6 kV, the sampling cone at 30 V and the extraction cone at 4.0 V. The scan time was 0.1 s covering the 50–1500 Da mass range with an interscan time of 0.02 s. The source temperature was 120°C and the desolvation temperature was set at 450°C. Nitrogen gas was used as the nebulization gas at a flow rate of 700 L/h and cone gas was added at 50 L/h. The software used to control the hyphenated system and do all data manipulation was MassLynx 4.2 (SCN 1028).

### 2.4 Antioxidant Enzymes Assays

#### 2.4.1 Plant Extraction

To determine antioxidant activity, 5 g of ground leaf powder (from each of the photon‐treated and untreated samples) was dissolved in 80% methanol in 50 mL Eppendorf tubes. The homogenate was centrifuged at 4500 rpm for 20 min using a Benchmark LC‐8 35008 Centrifuge (Sigma‐Aldrich) and the supernatant was placed on a shaker for 24 h. The extracts were serially diluted to achieve different concentration levels (250, 125, 62.5, 31.25, 15.63, 7.81, and 3.91 µg/mL). Ascorbic acid (10 mg/mL) was prepared and used as a positive control in all assays. The dried extracts were filtered using a Whatman filter paper and evaporated to dryness under reduced pressure using a rotary evaporator. Dried extracts were then stored in a closed cabinet at room temperature until use.

#### 2.4.2 SOD Assay

The activity of SOD in the maize leaf samples was measured using the SOD determination kit‐WST (Cat. no: 19160; Sigma‐Aldrich). The kit employs tetrazolium salt, WST‐1, which generates a water‐soluble formazan dye when reduced by superoxide anions. The 30 mg extract was weighed and added to 5 mL of DMSO (10%). Then, 1 mL of working solution (WST) was diluted with 19 mL of buffer solution. In each well of a 96‐well microplate, 20 µL of sample solution, 200 µL of the WST‐1 working solution and 20 µL of the enzyme working solution were combined. The mixture was then incubated at 37°C for 20 min to generate a water‐soluble formazan dye. The SOD activity was then determined by measuring the absorbance at 450 nm using a microplate reader, with 10 mg/mL of ascorbic acid serving as the positive control. The percentage of inhibition of absorbance at 450 nm was calculated using the equation given in the kit.

$$\begin{array}{ccc} & & \mathrm{SOD}\;\mathrm{activity}\;(\mathrm{inhibition}\;\mathrm{rate}\;\%)\\ & & \;=(A_{\mathrm{Blank}1}-A_{\mathrm{Blank}3})-(A_{\mathrm{Sample}}-A_{\mathrm{Blank}2})/(A_{\mathrm{Blank}1}-A_{\mathrm{Blank}3})\\ & & \;\times 100\% \end{array}$$

#### 2.4.3 CAT Assay

The activity of CAT was measured using the CAT assay kit (Cat. no: CAT100; Sigma‐Aldrich), which provides a colorimetric assay for CAT activity. The assay uses a substituted phenol (3,5‐dichloro‐2‐hydroxybenzene‐sulfonic acid), which is oxidatively coupled to 4‐aminoantipyrine in the presence of hydrogen peroxide and horseradish POD (HRP) to give a red quinoneimine dye that absorbs at 520 nm. The supernatant was incubated in the substrate solution, which contained H_2_O_2_ (75 µL/mL in 50 mM potassium phosphate buffer, pH 7.0). In all cases ultrapure water was used. Before use, the 2 mL enzyme dilution buffer (assay buffer) was diluted with 20 mL of water and kept at room temperature. Next, 1.45 mL of assay buffer was used to dissolve 1 mg of solid POD. Thirty milliliters of chromogen solution and 30 µL of POD solution were combined to create the color reagent. The absorbance was measured at 520 nm at room temperature.

#### 2.4.4 POD Assay

The POD activity was determined using a POD assay kit (Cat. no: MAK092; Sigma‐Aldrich). The manufacturer's instructions were followed to dilute the standards. In summary, a 0.1 mM standard solution was prepared by diluting approximately 10 µL of the 12.5 mM H_2_O_2_ solution with 1240 µL of test buffer. All samples and standards were run in duplicates. For fluorometric detection, 100 µL of 0.1 mM standard solution was diluted with 900 µL of the test buffer, to make a standard solution of 0.01 mM. Then the 0.01 mM standard solution was added (0, 10, 20, 30, 40, and 50 µL) into a 96‐well plate. For both the colorimetric and fluorometric assays, 50 µL of sample for each reaction (well) was added, respectively. The absorbance of each well was measured at 570 nm after it was incubated at room temperature for 5 min.

## 3 Results

### 3.1 The Effect of Photon on Metabolites of *Z. mays* Collected Between 1 and 24 h

**FIGURE 1:**
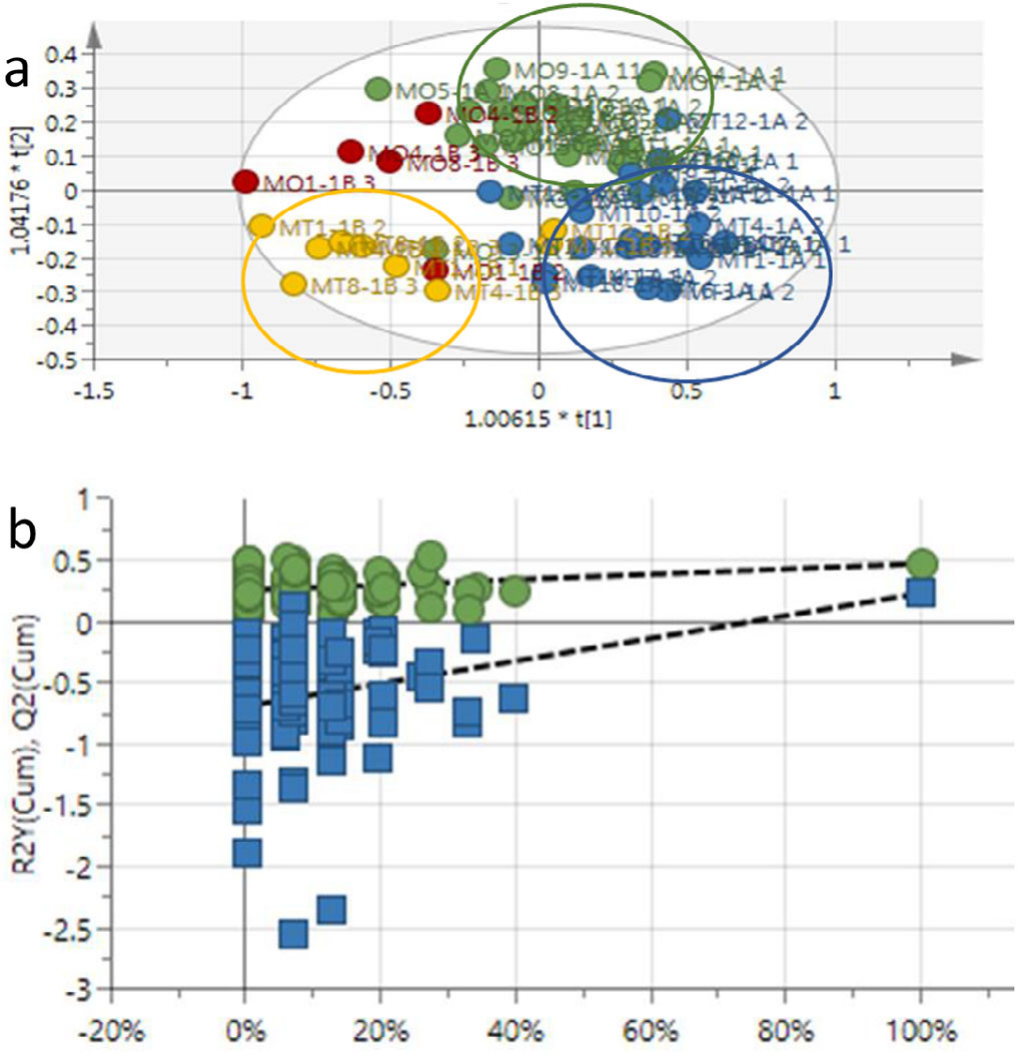
(a) The orthogonal partial least squares–discriminant analysis (OPLS‐DA) score plot of NMR methanol leaf extracts collected at 1 and 2 h posttreatment (blue—treated/1 h; green—untreated/1 h; yellow—treated/2 h; red—untreated/2 h). (b) OPLS‐DA model permutation test results (*n* = 100) where *R* ^2^ = (0.0, 0.28) and *Q* ^2^ = (0.0, −0.69).

The ^1^H‐NMR spectroscopy data of plant metabolic profiles was obtained to distinguish between treated and untreated *Z. mays* leaf extracts using SIMCA software. The supervised OPLS‐DA statistical model was then used to compare leaf extracts showing separation between the sample groups (blue = 1 h treated, green = 1 h untreated, yellow = 2 h treated, and red = 2 h untreated) within the 95% confidence interval in Figure 1a. Clear separation was observed for the 1 h treated (blue) and 1 h untreated (green) samples with slight overlap for the 2 h treated (yellow) and 2 h untreated (red) samples. The 2 h treated and untreated samples, however separated from each other in clusters. The *R* ^2^ *X* and *Q* ^2^ values represent the separation of samples and the model revealed goodness of fit (*R* ^2^ *X* = 0.975) and predictability (*Q* ^2^ = 0.641). Furthermore, to validate the model, the permutation test (*n* = 100) was performed. A permutation analysis and 100 permutation tests with *R* ^2^ = (0.0, 0.28) and *Q* ^2^ = (0.0, −0.69) were observed in Figure 1b.

**FIGURE 2:**
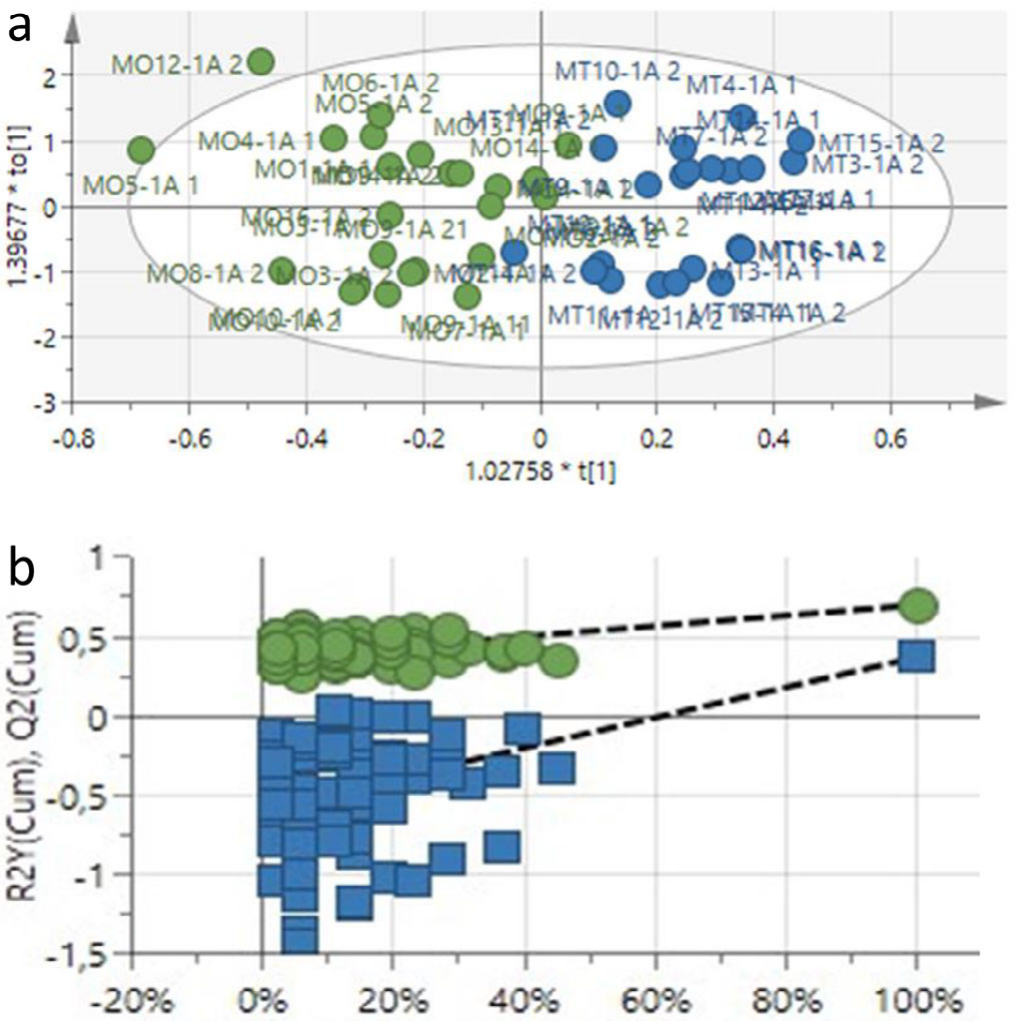
(a) The OPLS‐DA score plot of ^1^H‐NMR data from treated and untreated *Zea mays* leaf extracts collected after 1 h of photon application. Green = untreated, and blue = treated. (b) The ^1^H‐NMR permutation testing (*n* = 100 permutations) *R* ^2^ = (0.0, 0.37), *Q* ^2^ = (0.0, 0.583).

The OPLS‐DA score plot clearly separated the treated (blue) from untreated (green) leaf samples with a goodness of fit (*R* ^2^ *X* = 0.883) and predictability score value of *Q* ^2^ = 0.374 (Figure 2a). The separation of the samples implicated a distinct difference in the metabolite profile of the treated versus the untreated samples. The permutation test (*n* = 100) compares the *R* ^2^ and *Q* ^2^ values of the true model with the permuted mode. The model showed higher *R* ^2^ and *Q* ^2^ values compared to the permuted model (Figure 2b).

**FIGURE 3:**
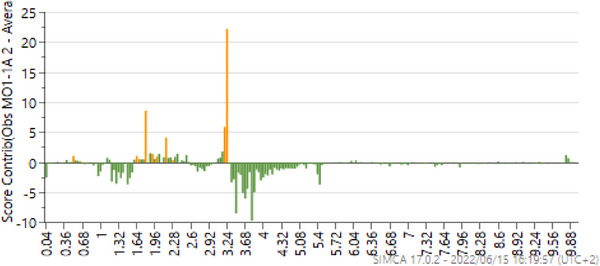
The contribution plot showing the NMR regions of the methanol treated and untreated leaf samples collected between 1 and 2 h. The NMR regions contributed to the separation of treated and untreated samples, with the bars above the line positively associated with the treated samples.

Figure 3 shows the contribution plot of samples collected between 1 and 2 h after application of photon. The contribution plot was used to determine the NMR regions of importance that contributed to the separation, with the positive bars associated with the treated samples. The focus was on these regions when the annotation of compounds was initiated, with most of the important NMR peaks in the aliphatic and early sugar regions. In addition, the OPLS‐DA permutation test (*n* = 100) is provided in Table S1.

### 3.2 The Metabolite Composition of the *Z. mays* Leaf Extracts Collected Between 1 and 3 Weeks After Photon Application

**FIGURE 4:**
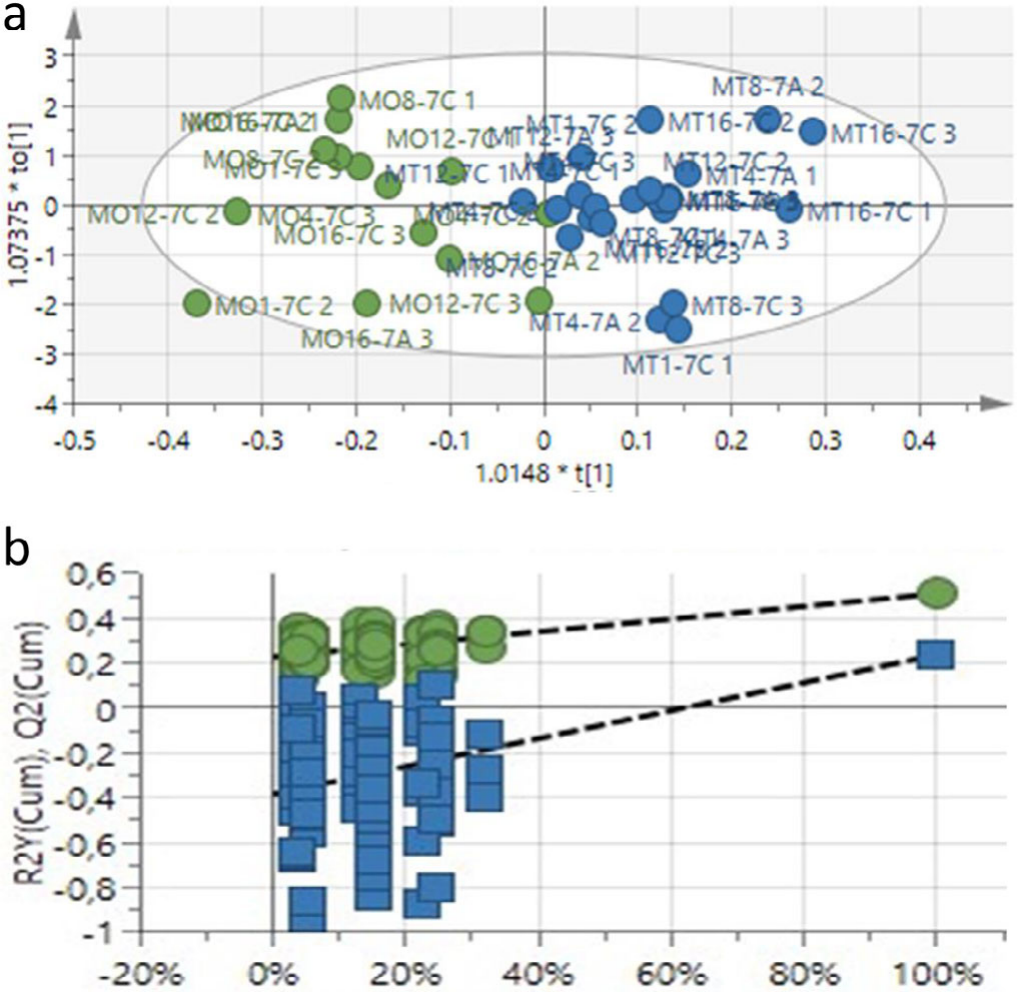
(a) The OPLS‐DA score plot of treated and untreated *Zea mays* leaf extracts collected between 1 and 3 weeks. Green = untreated, and blue = treated. (b) The OPLS‐DA model response permutation test (*n* = 100) corresponding to *y*‐axis intercepts *R* ^2^ = (0.0, 0.228), *Q* ^2^ = (0.0, −0,385).

To further demonstrate the duration of the effect of photon between treated and untreated leaf samples another OPLS‐DA score plot of samples collected between 1 and 3 weeks was generated. Figure 4a depicts the separation of treated (blue) samples from untreated (green) samples collected between 1 and 3 weeks. In congruence with the comparison with samples at 2 weeks, clear separation was obtained for the samples taken at 1 and 3 weeks after photon application. Based on the separation of the samples, it is also evident that the metabolite profile of the two groups is distinctly different. The model demonstrated goodness of fit and predictability, as indicated by *R* ^2^ *X* = 0.808, *R* ^2^ *Y* = 0.51, and *Q* ^2^ = 0.238, respectively. A response permutation test with (*n* = 100) was constructed to validate the predictive capability of the OPLS‐DA model (Figure 4b). The results validated the capacity of OPLS‐DA to differentiate between treated and untreated sample classes.

**FIGURE 5:**
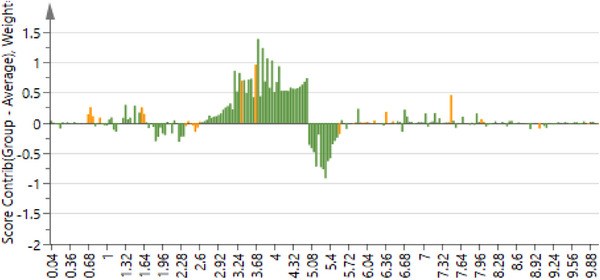
The contribution plot of the methanol‐treated and non‐treated maize leaf extracts collected between 1 and 3 weeks. The positive bars represent NMR regions that are positively associated with the treated samples.

Figure 5 shows the contribution plot of leaf extracts collected between 1 and 3 weeks. The contribution plot was used to determine the NMR regions of importance that contributed to the separation, with the positive bars associated with the treated samples. The focus was on these regions when the annotation of compounds was initiated. The sugar and aliphatic regions showed a positive association with the treated samples with a few individual peaks in the aromatic region.

Chenomx software was utilized to annotate compounds, and the values of NMR spectral regions were compared with NMR values from the HMDB to confirm the annotated compounds involved in stress tolerance. AzA and salicylic acid (SA) showed a significant increase in photon‐treated samples. Amino acids (4‐aminobutyrate [GABA], alanine, and asparagine), and sugars (maltose, sucrose, and trehalose) were also found in higher concentrations (Table 1). The results of this study also revealed higher concentrations of other compounds such as malate, aconitate and chlorogenic acid in photon‐treated maize plants after 12 h of treatment.

**TABLE 1:** The ^1^H‐NMR compounds annotated using Chenomx with their corresponding NMR peaks that contributed to the separation of treated and untreated methanolic leaf extracts of *Zea mays*.

Compound	^1^H‐NMR chemical shift (ppm)	Chenomx (ppm)	Human metabolite database	Literature
Salicylic acid^a^	7.0 7.54 7.85	7.0 7.5 7.8	7.82	
Azelaic acid^a^	1.54 1.29 2.1	1.5 1.3 2.1	1.29 1.3	
Alanine^a^	1.45	1.5 3.8	1.46	[18]
Asparagine^a^	2.65 2.9 4.0	2.8 2.9 4.0	2.84	[19]
Chlorogenic acid^a^	4.23 6.4 6.9 7.1 7.2 7.6	2.0 2.1 2.2 3.9 4.3 5.3 6.4 6.9 7.1 7.2 7.6	2.03 6.39 6.94 7.12 7.19 7.65	[20]
Malate	2.65 2.71 4.31	2.7 2.4 4.3	2.37 2.65 4.30	[21]
T‐aconitate	3.58 6.65	3.4 6.6	3.44 6.58	[19]
4‐Aminobutyric acid (GABA)^a^	1.9 2.1 2.29 3.0 3.18	1.9 2.3 3.0	1.89 2.29 3.0	[21]
Maltose^a^	3.89 4.6 5.2 5.4	3.9 4.6 5.2 5.4	3.84 5.22 5.40	[20]
Sucrose^a^	4.14 4.2 5.37	4.1 4.2 5.4	4.21 5.4	[22]
Trehalose^a^	5.16	5.2		[22]
Lactulose	3.85 3.92 4.0 4.02 4.35 4.52	3.6 4.0 4.4 4.5 4.6	3.63 3.85 4.05 4.06	[18]

^a^ High in treated plants.

LC–MS analysis was conducted to confirm the compounds annotated by the metabolomic analysis. Due to the polarity of most of the compounds, they co‐eluted early on the chromatogram and separation was not possible. Alanine, AzA, sucrose, and fructose were confirmed using an in‐house NIST database (Table S2).

### 3.3 Antioxidant Enzymes

**FIGURE 6:**
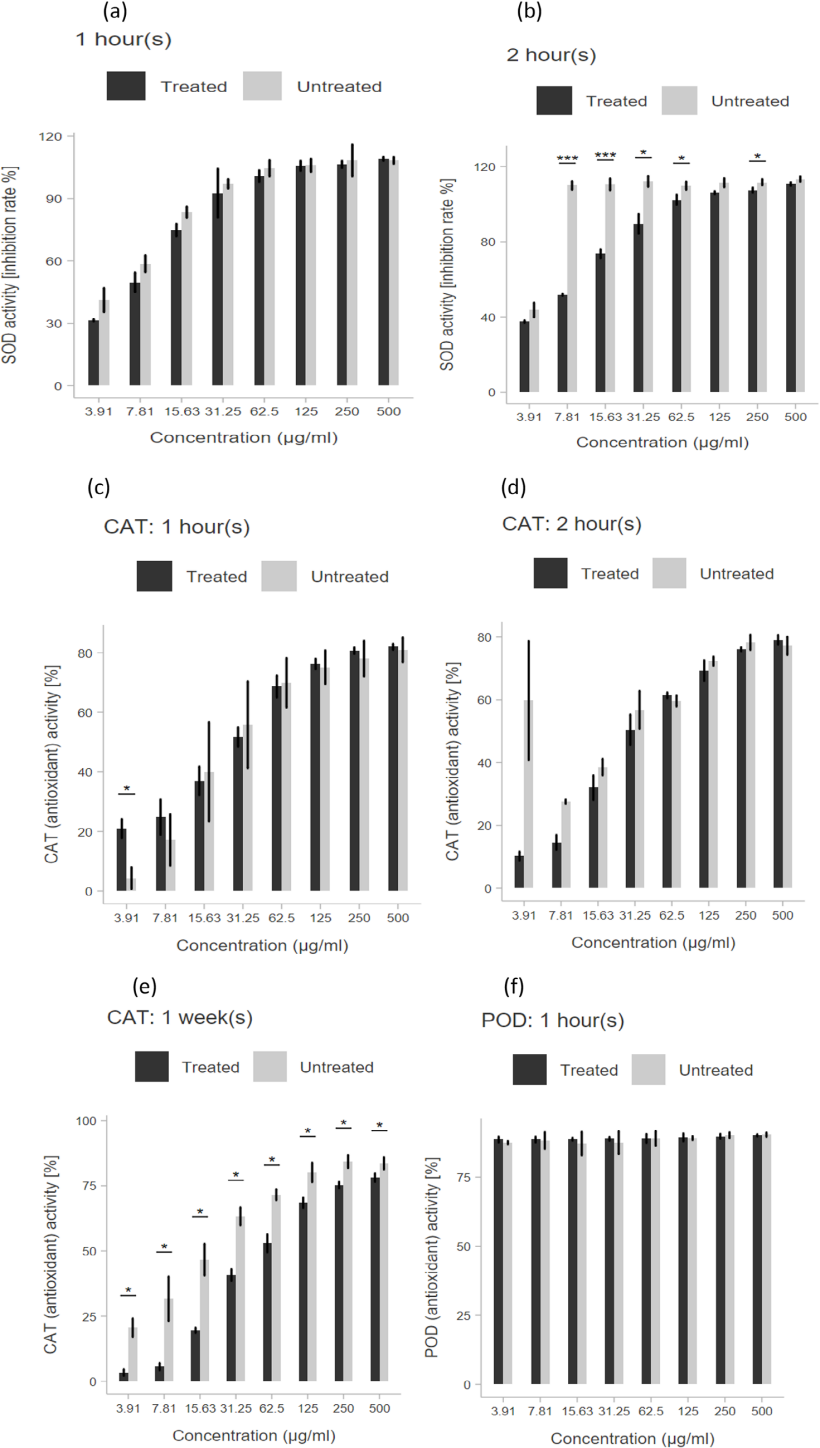
Effect of photon on the activities of SOD (a, b), CAT (c–e), and POD (f) enzyme activities of different concentrations of *Zea mays* leaf extracts. Black graphs—treated; gray—untreated. The bar graphs with asterisks *p ≤ 0.05, **p ≤ 0.01, ***p ≤ 0.001.

The leaf extracts of *Z. mays* demonstrated the highest activity of the SOD enzyme within the concentration range of 500 to 3.91 µg/mL. Higher (SOD) activity was achieved at all concentrations in untreated samples, although only significant after 2 h of treatment. The CAT activity (Figure 6b) increased at lower concentrations of 7.81 and 3.91 µg/mL for the treated samples. However, activity decreased over time after application and increased activity was observed in untreated samples (Figure 6c–e) with significant differences between treated and untreated in 1 week. No significant differences were observed between photon‐treated and untreated leaf extracts in the activity POD enzyme (Figure 6f).

## 4 Discussion

### 4.1 ^1^H‐NMR Analysis

In this study, the effect of exogenous application of photon on leaf extracts of *Z. mays* was compared using metabolomic analysis. Metabolite profiling of *Z. mays* was performed using ^1^H‐NMR spectroscopy. The metabolite annotation revealed the existence of several compounds from various classes. A total of 13 metabolites were detected including SA, AzA, amino acids (GABA, alanine, and asparagine), sugars (sucrose, maltose, and trehalose), malate, ascorbate, T‐aconitate, chlorogenic acid, and lactulose. All the compounds are in agreement with prior research [23] except AzA and SA. Among these annotated compounds, this study demonstrates that photon treatment resulted in a notable increase in AzA, SA, with a marked increase in malate, chlorogenic acid and maltose compared to the untreated maize leaf samples.

The SA content was enhanced 12 h posttreatment, reaching a value of 127.9 mM. SA has been reported to be associated with environmental stress responses in plants [24] and the induction of systemic acquired resistance (SAR) [25]. It improves drought resistance by controlling metabolic processes including regulation of stomata, photosynthesis, glucose metabolism, signal transduction, and generation of osmolytes and specialized metabolites [26]. Under drought stress, Degenkolbe et al. [27] found that SA is considerably higher in resistant rice cultivars than in susceptible ones, making it a possible marker of drought resistance. A previous study by Venegas‐Molina et al. [28] reported that different elicitors that were applied to Brassicaceae and *Arabidopsis* activated the SA‐dependent signaling pathway. According to Horváth et al. [29], SA is a component of the defensive signaling pathway in response to abiotic challenges such as osmotic and salt stress, temperature fluctuations, and toxic metal exposure.

In another study, SA has been reported to trigger major enzymes, including SOD POD, CA and APX, when exposed to various abiotic stress factors [30]. These enzymes are known to be affected by various abiotic and biotic stress factors, making them important regulators of SA functions [11], for example, under arsenic stress in *Glycine max* [31]. In a study conducted by Tahjib‐Ul‐Arif et al. [32], it was determined that an increase in CAT and APX activity is responsible for ROS scavenging in maize plants under salt‐stress. Previous research found that SA increased the salt tolerance of *Torreya grandis* by increasing the activity of antioxidant enzymes, which finally triggered the photosynthetic process and reduced oxidative stress [33]. In addition, Sakata et al. [34] reported that SA application increased the antioxidant capacity in rice. The high concentration of SA in treated plants suggests that photon is also responsible for an enhanced antioxidant response as a result of priming, although it was not observed in the enzyme assays used in this study.

AzA content in treated samples increased to 143.2 mM at 2 weeks, subsequently declining to 18.3 mM after 3 weeks. AzA is a natural signaling compound that induces plant defense response by means of a priming mechanism [35], and acts as a derivative of oleic acid, linoleic, and linolenic acid [36]. In addition, it functions as a mobile SAR signal in the vascular sap that confers both local and systemic resistance to biotic stress. These unsaturated fatty acids are known to play a key role in activating plant defense genes. Wang et al. [37] found that overexpression of AzA in *Arabidopsis* led to an increase in lignin buildup and an improvement in pathogen resistance. It is hypothesized that AzA can serve as an indicator of lipid peroxidation in plants subjected to biotic and abiotic stresses [38]. However, currently there is no published information on the mechanism of AzA in plants under abiotic stress conditions.

The NMR analysis also confirmed sucrose, maltose and trehalose in both treated and untreated samples, although higher concentrations were found in the treated samples. After 2 weeks of treatment, the maximum concentration of maltose detected by ^1^H‐NMR analysis was 82.6 mM. Following photon treatment, sucrose and trehalose exhibited reduced concentrations in comparison to maltose. It has been shown that plants accumulate soluble sugars as osmoprotectants during plant stress [39, 40]. In a comprehensive review, Cheng et al. [39] examined the function of trehalose in plant metabolism. This review reports that the levels of trehalose change in parallel with sucrose, which is the major product of photosynthesis and the main transport sugar in plants. Furthermore, it proposed the existence of a bidirectional network, in which trehalose is a signal of sucrose availability and acts to maintain sucrose concentrations within an appropriate range [41]. It has been demonstrated that these carbohydrates have a favorable effect on stress conditions and was similarly found in this study with an increase in the concentrations of these sugars in treated samples. Shi et al. [42] found that drought‐tolerant *Brachypodium* cultivars accumulated higher concentrations of soluble sugars (sucrose, trehalose, fructose, and galactose) than drought‐sensitive cultivars, providing beneficial effects in response to drought stress. SA was reported to inhibit sucrose and valine uptake in a concentration‐dependent manner. The high concentrations of maltose in maize plants show that a blend of dicarboxylic acids (photon) may be responsible for adjusting the osmotic environment of cells. It is important to acknowledge that the carbohydrate content in maize leaves may vary in response to environmental conditions.

The amino acids GABA, alanine and asparagine as well as malate and *trans*‐aconitate were present in both treated and non‐treated leaf extracts of *Z. mays*. A slight increase in concentration of these amino acids was detected between 12 and 24 h in treated samples but declined after 1 week and remained the same as in untreated samples. The content of various amino acids can vary depending on the variety of the plants and the amount of stress they experience [43]. Despite the presence of some amino acids in treated and untreated samples, photon treatment significantly increased their concentrations. These amino acids (GABA, alanine, and asparagine) are essential for plant metabolism, homeostasis, and serve as signaling compounds for biotic and abiotic stress tolerance. In plants, GABA is reported to act as a signal molecule under different environmental stresses and is mainly involved in growth and development through the GABA shunt, a bypass of the TCA cycle [44]. The increase in asparagine has been reported to improve salt tolerance in a C4 plant *Aeluropus lagopoide* [45]. This finding agrees with the findings of Gavaghan et al. [19] which showed an increase in tolerance to salt stress in maize caused by various amino acids, including asparagine, alanine, GABA, and glutamate. In the present investigation, the concentrations of amino acids were found to be slightly higher in the treated samples. It is possible that the plants subjected to stressful conditions, might show a more significant increase in these annotated metabolites.

Furthermore, chlorogenic acid was detected in both treated and untreated plants, with higher amounts in treated samples from 12 h to 2 weeks after treatment. The involvement of chlorogenic acid in environmental stress tolerance in plants is well‐documented, as it supports the stress‐responsive mechanisms that enable plants to counteract the detrimental impacts of stress [46]. Under water stress, it has been reported to enhance antioxidant capacity in *Achillea pachycephala* [47].

Metabolomics analysis therefore showed a comprehensive stress response in treated maize plants by elevating various sugars and amino acids, as well as chlorogenic acid due to priming with photon.

### 4.2 Enzyme Assays

According to Chaves et al. [48], the defensive mechanisms at the leaf level need to be activated rapidly to prevent disruption of photosynthetic mechanisms. In the present study, leaf extracts of *Z. mays* collected at 1 h, 2 h, 12 h, 24 h, 1 week, 2 weeks, and 3 weeks were screened for their inhibitory activity using SOD, CAT, and POD enzymes following photon application. The results show that POD was not activated by treatment in any samples. CAT was only increased at lower concentrations (7.81 and 3.91 µg/mL) but declined over time so that higher CAT activity was generally observed in untreated samples. A study in exogenous application treatment of SA as priming and foliar spray revealed increases in SOD, CAT, POD, and APX activity in maize leaves at different growth stages (V6, V12, R2) [49]. Ning et al. [49] further observed a reduction in yield by drought stress at R2 stage compared to V6 and V12.

The present study showed contrasting results with a decrease in SOD and CAT activities at 24 h in photon‐treated plants, which was below the levels of untreated extracts. Untreated extracts exhibited significantly higher CAT enzyme activity than treated extracts at 1 week for all concentrations tested. Generally, a better activity of CAT was observed at 125–500 µg/mL which was almost the same as that of the positive control. Furthermore, all leaf extracts of *Z. mays* tested at various concentrations exhibited better POD activity than CAT, with no statistically significant distinction between treated and untreated. Zeng et al. [50] found that CAT was the major enzyme that eliminated H_2_O_2_ in soybeans at high concentrations, exceeding POD. POD activity was, however, found to be elevated at low concentrations. In the current study, the enzymes investigated that photon has a minimal impact on the enzymes studied and that other antioxidant enzymes or systems are activated by elevated AzA and SA after priming.

## 5 Conclusion

In this study, a number of compounds were annotated that increased posttreatment. The NMR‐based metabolomics analysis demonstrated a substantial accumulation of SA and AzA in response to photon application compared to those of untreated plants. Since SA is known to increase both enzyme‐based and non‐enzyme‐based antioxidants, the large increase in these compounds supports the activation of a stress‐related system.

With SA being one of the major compounds identified in photon‐treated maize plants, the major antioxidant enzymes were further evaluated. The findings indicated that the use of photon on maize leaves as a priming agent does not have major effects on SOD, CAT, and POD. The involvement of other antioxidant enzymes with enhanced levels of amino acids (GABA, alanine, and asparagine), sugars (maltose, sucrose, and trehalose), and compounds such as chlorogenic acid indicate that various protective mechanisms have been activated in the maize plants, resulting in an effective response to stress protection after priming with dicarboxylic acids (photon). In the future, stress applications should be examined to determine the impact of dicarboxylic acids (photon) on the stress response of maize.

## Conflicts of Interest

The authors declare no conflicts of interest.

## Supporting information




**Supporting File**: cbdv70204‐sup‐0001‐SuppMat.pdf

## Data Availability

The data that support the findings of this study are available from the corresponding author upon reasonable request.

## References

[1] D. B. Medeiros , Y. Brotman , and A. R. Fernie , “The Utility of Metabolomics as a Tool to Inform Maize Biology,” Plant Communations 2 (2021): 100187, 10.1016/j.xplc.2021.100187. PMC8299083 34327322

[2] R. Guo , R. Qian , L. Yang , et al., “Interactive Effects of Maize Straw‐Derived Biochar and N Fertilization on Soil Bulk Density and Porosity, Maize Productivity and Nitrogen Use Efficiency in Arid Areas,” Journal of Soil Science and Plant Nutrition 22 (2022): 4566–4586, 10.1007/s42729-022-00881-1.

[3] T. Isah , “Stress and Defense Responses in Plant Secondary Metabolites Production,” Biological Research 52 (2019): 39, 10.1186/s40659-019-0246-3. 31358053 PMC6661828

[4] L. Yang , K. S. Wen , X. Ruan , Y. X. Zhao , F. Wei , and Q. Wang , “Response of Plant Secondary Metabolites to Environmental Factors,” Molecules 23 (2018): 762, 10.3390/molecules23040762. 29584636 PMC6017249

[5] A. Humbal and B. Pathak , “Influence of Exogenous Elicitors on the Production of Secondary Metabolite in Plants: A Review (“VSI: Secondary Metabolites”),” Plant Stress 8 (2023): 100166, 10.1016/j.stress.2023.100166.

[6] A. Ramakrishna and G. A. Ravishankar , “Influence of Abiotic Stress Signals on Secondary Metabolites in Plants,” Plant Signaling & Behavior 6, no. 11 (2011): 1720–1731, 10.4161/psb.6.11.17613. 22041989 PMC3329344

[7] B. Onwuka and B. Mang , “Effects of Soil Temperature on Some Soil Properties and Plant Growth,” Advances in Plants & Agriculture Research 8 (2018): 34–37, 10.15406/apar.2018.08.00288.

[8] T. Obata , “Metabolons in Plant Primary and Secondary Metabolism,” Phytochemistry Reviews 18, no. 6 (2019): 1483–1507, 10.1007/s11101-019-09619-x.

[9] S. Rasmann , M. De Vos , C. L. Casteel , et al., “Herbivory in the Previous Generation Primes Plants for Enhanced Insect Resistance,” Plant Physiology 158, no. 2 (2012): 854–863, 10.1104/pp.111.187831. 22209873 PMC3271773

[10] S. M. Westman , K. J. Kloth , J. Hanson , A. B. Ohlsson , and B. R. Albrectsen , “Defence Priming in Arabidopsis—A Meta‐Analysis,” Scientific Reports 9 (2019): 13309, 10.1038/s41598-019-49811-9. 31527672 PMC6746867

[11] N. Mishra , C. Jiang , L. Chen , A. Paul , A. Chatterjee , and G. Shen , “Achieving Abiotic Stress Tolerance in Plants Through Antioxidative Defense Mechanisms,” Frontiers in Plant Science 14 (2023): 1110622, 10.3389/fpls.2023.1110622. 37332720 PMC10272748

[12] Crop Microclimate Management . “Methods for Increasing Tolerance to Abiotic Stress in Plants” US Patent 8,846,573 (U.S. Patent and Trademark Office, 2014).

[13] C. Brunetti , R. M. George , M. Tattini , K. Field , and M. P. Davey , “Metabolomics in Plant Environmental Physiology,” Journal of Experimental Botany 64, no. 13 (2013): 4011–4020, 10.1093/jxb/ert244. 23922358

[14] M. Giera , O. Yanes , and G. Siuzdak , “Metabolite Discovery: Biochemistry's Scientific Driver,” Cell Metabolism 34, no. 1 (2022): 21–34, 10.1016/j.cmet.2021.11.005. 34986335 PMC10131248

[15] T. Obata and A. R. Fernie , “The Use of Metabolomics to Dissect Plant Responses to Abiotic Stresses,” Cellular and Molecular Life Sciences 69, no. 19 (2012): 3225–3243, 10.1007/s00018-012-1091-5. 22885821 PMC3437017

[16] H. M. Heyman and J. J. M. Meyer , “NMR‐Based Metabolomics as a Quality Control Tool for Herbal Products,” South African Journal of Botany 82 (2012): 21–32, http://hdl.handle.net/2263/58487.

[17] S. Osorio and J. G. Vallarino , Metabolite Profiling in Plants (John Wiley & Sons, Ltd., 2017), 10.1002/97804780470015902.0020105.pub2.

[18] M. T. Akhtar , M. Samar , A. A. Shami , et al., “ ^1^H‐NMR‐Based Metabolomics: An Integrated Approach for the Detection of the Adulteration in Chicken, Chevon, Beef and Donkey Meat,” Molecules 26, no. 15 (2021): 4643, 10.3390/molecules26154643. 34361796 PMC8347375

[19] C. L. Gavaghan , J. V. Li , S. T. Hadfield , et al., “Application of NMR‐Based Metabolomics to the Investigation of Salt Stress in Maize (*Zea mays*),” Phytochemical Analysis 22 (2011): 214–224, 10.1002/pca.1268. 21204151

[20] N. Nkobole and G. Prinsloo , “ ^1^H‐NMR and LC‐MS Based Metabolomics Analysis of Wild and Cultivated *Amaranthus* spp,” Molecules 26, no. 4 (2021): 795, 10.3390/molecules26040795. 33557008 PMC7913636

[21] F. Gao , “1H NMR‐Based Metabolomics to Identify Resistance‐Related Metabolites in *Astragalus membranaceus* var. *mongholicus* Against *Fusarium* Root rot,” International Journal of Agriculture and Biology 26 (2021): 69–78.

[22] J. Joshi , G. Hasnain , T. Logue , et al., “A Core Metabolome Response of Maize Leaves Subjected to Long‐Duration Abiotic Stresses,” Metabolites 11 (2021): 797, 10.3390/metabo11110797. 34822455 PMC8625080

[23] P. Vasmatkar , K. Kaur , P. P. S. Pannu , G. Kaur , and H. Kaur , “Unraveling the Metabolite Signatures of Maize Genotypes Showing Differential Response Towards Southern Corn Leaf Blight by ^1^H‐NMR and FTIR Spectroscopy,” Physiological and Molecular Plant Pathology 108 (2019): 101441, 10.1016/j.pmpp.2019.101441.

[24] J. Liu , G. Qiu , C. Liu , et al., “Salicylic Acid, a Multifaceted Hormone, Combats Abiotic Stresses in Plants,” Life 12, no. 6 (2022): 886, 10.3390/life12060886. 35743917 PMC9225363

[25] D. Tripathi , G. Raikhy , and D. Kumar , “Chemical Elicitors of Systemic Acquired Resistance—Salicylic Acid and Its Functional Analogs,” Current Plant Biology 17 (2019): 48–59, 10.1016/j.cpb.2019.03.002.

[26] M. I. R. Khan , M. Fatma , T. S. Per , N. A. Anjum , and N. A. Khan , “Salicylic Acid‐Induced Abiotic Stress Tolerance and Underlying Mechanisms in Plants,” Frontiers in Plant Science 6 (2015): 462, 10.3389/fpls.2015.00462. 26175738 PMC4485163

[27] T. Degenkolbe , P. T. Do , J. Kopka , E. Zuther , D. K. Hincha , and K. I. Köhl , “Identification of Drought Tolerance Markers in a Diverse Population of Rice Cultivars by Expression and Metabolite Profiling,” PLoS ONE 8, no. 5 (2013): e63637, 10.1371/journal.pone.0063637. 23717458 PMC3661581

[28] J. Venegas‐Molina , S. Proietti , J. Pollier , W. Orozco‐Freire , D. Ramirez‐Villacis , and A. Leon‐Reyes , “Induced Tolerance to Abiotic and Biotic Stresses of Broccoli and *Arabidopsis* After Treatment With Elicitor Molecules,” Scientific Reports 10 (2020): 10319, 10.1038/s41598-020-67074-7. 32587286 PMC7316721

[29] E. Horváth , G. Szalai , and T. Janda , “Induction of Abiotic Stress Tolerance by Salicylic Acid Signaling,” Journal of Plant Growth Regulation 26, no. 3 (2007): 290–300, 10.1007/s00344-007-9017-4.

[30] H. Torun , O. Novák , J. Mikulík , A. Pěnčík , M. Strnad , and F. A. Ayaz , “Timing‐Dependent Effects of Salicylic Acid Treatment on Phytohormonal Changes, ROS Regulation, and Antioxidant Defense in Salinized Barley (*Hordeum vulgare* L.),” Scientific Reports 10 (2020): 13886, 10.1038/s41598-020-70807-3. 32807910 PMC7431421

[31] V. Chandrakar , A. Dubey , and S. Keshavkant , “Modulation of Antioxidant Enzymes by Salicylic Acid in Arsenic Exposed *Glycine max* L.,” Journal of Soil Science and Plant Nutrition 16, no. 3 (2016): 662–676, 10.4067/S0718-95162016005000048.

[32] M. Tahjib‐Ul‐Arif , M. N. Siddiqui , A. A. M. Sohag , et al., “Salicylic Acid‐Mediated Enhancement of Photosynthesis Attributes and Antioxidant Capacity Contributes to Yield Improvement of Maize Plants Under Salt Stress,” Journal of Plant Growth Regulation 37 (2018): 1318–1330, 10.1007/s00344-018-9867-y.

[33] T. Li , Y. Hu , X. Du , H. Tang , C. Shen , and J. Wu , “Salicylic Acid Alleviates the Adverse Effects of Salt Stress in *Torreya grandis* cv. *merrillii* Seedlings by Activating Photosynthesis and Enhancing Antioxidant Systems,” PLoS ONE 9, no. 10 (2014): e109492, 10.1371/journal.pone.0109492. 25302987 PMC4193794

[34] T. Sakata , T. Oshino , S. Miura , et al., “Auxins Reverse Plant Male Sterility Caused by High Temperatures,” Proceedings National Academy of Science of the United States of America 107, no. 19 (2010): 8569–8574, 10.1073/pnas.1000869107. PMC2889339 20421476

[35] F. Araniti , E. Talarico , M. L. Madeo , et al., “Short‐Term Exposition to Acute Cadmium Toxicity Induces the Loss of Root Gravitropic Stimuli Perception Through PIN2‐Mediated Auxin Redistribution in *Arabidopsis thaliana* (L.) Heynh,” Plant Science 332 (2023): 111726, 10.1016/j.plantsci.2023.111726. 37149227

[36] J. Shah and J. Zeier , “Long‐Distance Communication and Signal Amplification in Systemic Acquired Resistance,” Frontiers in Plant Science 22, no. 4 (2013): 30, 10.3389/fpls.2013.00030. PMC3579191 23440336

[37] X. Y. Wang , D. Z. Li , Q. Li , et al., “Metabolomic Analysis Reveals the Relationship Between AZI1 and sugar signaling in Systemic acquired Resistance of Arabidopsis,” Plant Physiology and Ciochemistry 107 (2016): 273–287, 10.1016/j.plaphy.2016.06.016. 27337039

[38] A. L. Adam , G. Katay , A. Kunstler , and L. Kiraly , “Detection of Lipid Peroxidation‐Derived Free Azelaic Acid, a Biotic Stress Marker and Other Dicarboxylic Acids in Tobacco by Reversed‐Phase HPLC‐MS Under Non‐Derivatized Conditions,” in Reactive Oxygen Species in Plants: Methods in Molecular Biology, ed. A. Mhamdi (Humana Press, 2022), 191–200, 10.1007/978-1-0716-2469-2_14. 35657521

[39] Y. J. Cheng , S. H. Yang , and C. S. Hsu , “Synthesis of Conjugated Polymers for Organic Solar Cell Applications,” Chemical Reviews 109 (2009): 5868–5923, 10.1021/cr900182s. 19785455

[40] A. Panda , J. Rangani , and A. K. Parida , “Unraveling Salt Responsive Metabolites and Metabolic Pathways Using Non‐Targeted Metabolomics Approach and Elucidation of Salt Tolerance Mechanisms in the Xero‐Halophyte *Haloxylon salicornicum* ,” Plant Physiology and Biochemistry 158 (2021): 284–296, 10.1016/j.plaphy.2020.11.012. 33239222

[41] J. E. Lunn , I. Delorge , C. M. Figueroa , P. Van Dijck , and M. Stitt , “Trehalose Metabolism in Plants,” Plant Journal 79 (2014): 544–567, 10.1111/tpj.12509. 24645920

[42] H. Shi , T. Ye , B. Song , X. Qi , and Z. Chan , “Comparative Physiological and Metabolomic Responses of Four *Brachypodium distachyon* Varieties Contrasting in Drought Stress Resistance,” Acta Physiologiae Plantarum 37 (2015): 122, 10.1007/s11738-015-1873-0.

[43] M. Trovato , D. Funck , G. Forlani , S. Okumoto , and R. Amir , “Editorial: Amino Acids in Plants: Regulation and Functions in Development and Stress Defense,” Frontiers in Plant Science 12 (2021): 772810, 10.3389/fpls.2021.772810. 34733310 PMC8559698

[44] L. Li , N. Dou , H. Zhang , and C. Wu , “The Versatile GABA in Plants,” Plant Signaling & Behavior 16, no. 3 (2021): 1862565, 10.1080/15592324.2020.1862565. 33404284 PMC7889023

[45] H. Sobhanian , N. Motamed , F. R. Jazii , T. Nakamura , and S. Komatsu , “Salt Stress Induced Differential Proteome and Metabolome Response in the Shoots of *Aeluropus lagopoides* (Poaceae), a Halophyte C_4_ Plant,” Journal of Proteome Research 9 (2010): 2882–2897, 10.1021/pr900974k. 20397718

[46] D. R. J. Soviguidi , R. Pan , Y. Liu , L. Rao , W. Zhang , and X. Yang , “Chlorogenic Acid Metabolism: The Evolution and Roles in Plant Response to Abiotic Stress,” Phyton—International Journal of Experimental Botany 91, no. 2 (2021): 239–255, 10.32604/phyton.2022.018284.

[47] M. Hodaei , M. Rahimmalek , A. Arzani , and M. Talebi , “The Effect of Water Stress on Phytochemical Accumulation, Bioactive Compounds and Expression of Key Genes Involved in Flavonoid Biosynthesis in *Chrysanthemum morifolium* L,” Industrial Crops and Products 120 (2018): 295–304, 10.1016/j.indcrop.2018.04.073.

[48] M. M. Chaves , J. Flexas , and C. Pinheiro , “Photosynthesis Under Drought and Salt Stress: Regulation Mechanisms From Whole Plant to Cell,” Annals of Botany 103, no. 4 (2009): 551–560, 10.1093/aob/mcn125. 18662937 PMC2707345

[49] D. Ning , X. Li , L. Li , et al., “Analyses of Physio‐Chemical and Transcriptomic Revealed the Mechanisms of Forliar Application of Silicon Enhancing Drought Resistance in Maize (zea mays L.),” 10.2139/ssrn.5234948.

[50] C. Q. Zeng , W. X. Liu , J. Y. Hao , et al., “Measuring the Expression and Activity of the CAT Enzyme to Determine Al Resistance in Soybean,” Plant Physiology and Biochemistry 144 (2019): 254–263, 10.1016/j.plaphy.2019.09.026. 31593898

