# Supplementary material for: The Effect of Exogenously Applied Dicarboxylic Acids (Photon) on the Maize (Zea mays) Metabolite Profile
Source: Chem Biodivers. 2025 Jul 22;22(11):e01243. doi: 10.1002/cbdv.202501243 (PMC12629178; doi:10.1002/cbdv.202501243)
Supplement: Supplementary file 1 — Supporting File: cbdv70204‐sup‐0001‐SuppMat.pdf [file CBDV-22-e01243-s001.pdf]

Table 1: The OPLS_DA model quality for samples collected after 1 hour and 2 hours following photon treatment.

**OPLS-DA permutation test (n=100)**				
R^2X	R^2Y	Q^2	R^2	Y^2
0.97	0.55	0.64	(0.0, 028)	(0.0, -69)

Table 2: Compounds confirmed and identified on LC-MS

**Compound**	**Mass**	**Calc. mass**	**mDa**	**PPM**	**DBE**	**i-FIT**	**Norm**	**Conf (%)**	**Formula**
Alanine	88.04003	88.0399	0.4	4.5	1.5	542.9	n/a	n/a	$C_3H_6NO_2$
Azelaic acid	187.0976	187.0970	0.6	3.2	2.5	871.4	n/a	n/a	$C_9H_{15}O_4$
Sucrose	341.1084	341.1048	0.0	0.0	2.5	176.1	n/a	n/a	$C_{12}H_{21}O_{11}$

Table 2: Concentrations for annotated metabolites in mM from Chenomx NMR Suite(**C**=Untreated: **T**-treated).

**Compound**	**1 h**	**2 h**	**12 h**	**24 h**	**1 week**	**2 weeks**	**3 weeks**
**Salicylic acid**	C=8.0515<br>T=61.2668	C=61.8920<br>T=99.6389	C=66.1391<br>T=100.9292	C=88.7063<br>T=118.3719	C=89.5631<br>T=127.8851	C=69.5948<br>T=80.6585	C=71.0298<br>T=97.6599
**Azelaic acid**	C=2.3874<br>T=18.3070	C=75.4871<br>T=88.9191	C=36.0912<br>T=92.8909	C=28.5654<br>T=102.3415	C=42.2524<br>T=112.2540	C=29.2702<br>T=143.2366	C=23.2236<br>T=38.2362
**Malate**	C=5.7930<br>T=61.2262	C=110.3014<br>T=123.5443	C=121.2196<br>T=122.2546	C=35.9970<br>T=98.9431	C=63.0245<br>T=110.2171	C=73.2222<br>T=76.6454	C=68.0110<br>T=99.5329
**Asparagine**	C=5.3402<br>T=64.7817	C=145.5256<br>T=176.2590	C=128.1791<br>T=131.3983	C=38.9255<br>T=61.5405	C=63.7282<br>T=66.7366	C=78.0129<br>T=76.9525	C=73.6290<br>T=72.3424
**Ascorbate**	C=2.8260<br>T=36.6614	C=234.1769<br>T=61.6628	C=72.6089<br>T=72.6191	C=21.3203<br>T=33.6399	C=36.4304<br>T=60.8625	C=42.9346<br>T=44.2433	C=40.1966<br>T=59.2993
**T-aconitate**	C=1.4088<br>T=17.5528	C=27.5498<br>T=29.5153	C=27.7622<br>T=21.5368	C=13.4319<br>T=24.0816	C=28.4138<br>T=17.2553	C=21.7288<br>T=24.1136	C=19.1793<br>T=22.0070
**GABA**	C=5.5651<br>T=65.3455	C=35.0102<br>T=150.1994	C=103.4500<br>T=78.6347	C=48.7456<br>T=57.4160	C=76.1648<br>T=79.4757	C=65.1742<br>T=83.7884	C=69.3121<br>T=80.8904
**Lactulose**	C=2.8403<br>T=38.6958	C=62.3188<br>T=59.0827	C=61.1081<br>T=60.1731	C=30.9460<br>T=35.8414	C=70.6747<br>T= 36.6314	C=43.6219<br>T=40.5819	C=44.5228<br>T=49.5752
**Alanine**	C=5.2077<br>T=67.0641	C=71.1276<br>T=91.6889	C=77.8603<br>T=1.6492	C=31.6506<br>T=5.5282	C=53.6971<br>T=2.1491	C=33.5815<br>T=13.8375	C=46.1253<br>T=1.7569
**Chlorogenic acid**	C=12.6613<br>T=3.5342	C=20.0203<br>T=60.4345	C= 41.0204<br>T=82.3502	C=50.3134<br>T=91.0502	C=52.2335<br>T=92.0233	C=43.1245<br>T=44.3201	C=40.9124<br>T=41.2203
**Sucrose**	C=5.4381<br>T=8.3421	C=18.2301<br>T=50.5432	C= 28.2013<br>T=70.5403	C=39.8104<br>T=89.0201	C=44.3213<br>T=96.2403	C=41.1324<br>T=42.5403	C=38.2901<br>T=40.0135

**Maltose**	C=4.2307<br>T=5.0124	C=15.4532<br>T=31.0346	**C**= 21.0854<br>**T**=42.0543	**C**=33.0328<br>**T**=50.0304	**C**=35.0301<br>**T**=60.5043	C=30.4012<br>T=59.0230	C=28.0450<br>T=34.0454
**Trehalose**	C=2.0125<br>T=5.0345	C=12.0343<br>**T**=22.5903	**C**= 18.4056<br>**T**=41.0450	**C**=21.6504<br>**T**=58.045	**C**=22.0543<br>**T**=60.7408	C=19.4083<br>T=51.0345	C=14.4056<br>T=37.9023
